# Gender Differences in Idiopathic Pulmonary Fibrosis: Are Men and Women Equal?

**DOI:** 10.3389/fmed.2021.713698

**Published:** 2021-08-05

**Authors:** Lucile Sesé, Hilario Nunes, Vincent Cottin, Dominique Israel-Biet, Bruno Crestani, Stephanie Guillot-Dudoret, Jacques Cadranel, Benoit Wallaert, Abdellatif Tazi, Bernard Maître, Gregoire Prévot, Sylvain Marchand-Adam, Sandrine Hirschi, Sandra Dury, Violaine Giraud, Anne Gondouin, Philippe Bonniaud, Julie Traclet, Karine Juvin, Raphael Borie, Zohra Carton, Olivia Freynet, Thomas Gille, Carole Planès, Dominique Valeyre, Yurdagül Uzunhan

**Affiliations:** ^1^AP-HP, Service de Physiologie, Hôpital Avicenne, Bobigny, France; ^2^Centre Constitutif de Référence des Maladies Pulmonaires Rares, AP-HP, Service de Pneumologie, Hôpital Avicenne, Bobigny, France; ^3^EPAR, IPLESP UMR-S 1136, INSERM et Sorbonne Université, Paris, France; ^4^INSERM UMR 1272 “Hypoxia and the Lung,” Université Sorbonne Paris Nord, Bobigny, France; ^5^Centre Coordonnateur de Référence des Maladies Pulmonaires Rares, Hôpital Louis Pradel, Hospices Civils de Lyon, Université Lyon 1, Université de Lyon, INRAE, OrphaLung, Member of Respifil, ERN-LUNG, Lyon, France; ^6^Centre de Compétence des Maladies Pulmonaires Rares, AP-HP, Service de Pneumologie, Hôpital HEGP, Paris, France; ^7^Centre Constitutif de Référence des Maladies Pulmonaires Rares AP-HP, Service de Pneumologie, Hôpital Bichat, Paris, France; ^8^Centre de Compétence des Maladies Pulmonaires Rares, Service de Pneumologie, Hôpital Pontchaillou, Rennes, France; ^9^Centre Constitutif de Référence des Maladies Pulmonaires Rares, AP-HP, Service de Pneumologie, Hôpital Tenon and Sorbonne University, Paris, France; ^10^Centre Constitutif de Référence des Maladies Pulmonaires Rares, Service de Pneumologie, Hôpital Albert Calmette, Lille, France; ^11^Université de Paris, Centre de Référence National des Histiocytoses, AP-HP, Service de Pneumologie, Hôpital Saint-Louis, Paris, France; ^12^AP-HP, Service de Pneumologie, Hôpital Henri-Mondor, Créteil, France; ^13^Service de Pneumologie, Hôpital Larrey, Toulouse, France; ^14^Centre de Compétence des Maladies Pulmonaires Rares, Service de Pneumologie, Hôpital Bretonneau, Tours, France; ^15^Centre de Compétence des Maladies Pulmonaires Rares, Service de Pneumologie, Nouvel Hôpital Civil, Strasbourg, France; ^16^Centre de Compétence des Maladies Pulmonaires Rares, Service de Pneumologie, Hôpital Maison Blanche, Reims, France; ^17^AP-HP, Service de Pneumologie, Hôpital Ambroise Paré, Boulogne, France; ^18^Centre de Compétence des Maladies Pulmonaires Rares, Service de Pneumologie, Hôpital Jean Minjoz, Besançon, France; ^19^Centre Constitutif de référence des Maladies Pulmonaires Rares, Service de Pneumologie, Centre Hospitalier Universitaire Dijon Bourgogne, Dijon, France

**Keywords:** idiopathic pulmonary fibrosis, gender differences, occupational exposures, lung transplantation, women

## Abstract

**Background:** Idiopathic pulmonary fibrosis (IPF) is characterized by a male predominance. The aim of the study was to explore gender differences in a well-designed French multicentre prospective IPF cohort (COhorte FIbrose, COFI) with a 5-year follow-up.

**Methods:** Between 2007 and 2010, 236 patients with incident IPF were included in COFI. Gender characteristics were compared using a *t*-test, Chi-squared test and ANOVA, as appropriate. Survival analyses were performed.

**Results:** Fifty-one (22%) females and 185 (78%) males with an average age at diagnosis of 70.1 ± 9.20 and 67.4 ± 10.9 years, respectively, were included in the cohort. Women were significantly less exposed to tobacco smoke [never *n* = 32 (62.7%) vs. *n* = 39 (21.1%), *p* < 0.001] and to occupational exposure [*n* = 7 (13.7%) vs. *n* = 63 (34.1%), *p* = 0.012]. Baseline forced vital capacity, % of predicted (FVC%) was significantly better in women compare to men (83.0% ± 25.0 v. 75.4% ± 18.7 *p* = 0.046). At presentation honeycombing and emphysema on CT scan were less common in women [*n* = 40 (78.4%) vs. *n* = 167 (90.3%) *p* = 0.041] and [*n* = 6 (11.8%) vs. *n* = 48 (25.9%) *p* = 0.029], respectively. During follow-up fewer women were transplanted compared to men [*n* = 1 (1.96%) vs. *n* = 20 (10.8%) *p* = 0.039]. Medians of survival were comparable by gender [31 months (CI 95%: 28–40) vs. 40 months (CI 95%: 33–72) *p* = 0.2]. After adjusting for age and FVC at inclusion, being a woman was not associated to a better survival.

**Conclusions:** Women appear to have less advanced disease at diagnosis, maybe due to less exposure history compare to men. Disease progression and overall survival remains comparable regardless gender, but women have less access to lung transplantation.

## Introduction

Idiopathic pulmonary fibrosis (IPF) is a disease with a male predominance. In international cohorts, males account for ~70% of all IPF cases (1). A recent study highlighted that respiratory physicians rarely assigned IPF diagnosis in women and that gender was the most discriminating pre-test diagnostic probability criterion according to them (2). However, few studies analyzed gender-related features and outcomes in IPF. The aim of the study was to explore gender differences in a well-designed IPF cohort.

## Methods

Patients were selected from the French national multicentre prospective cohort (COhorte FIbrose, COFI). Patients were included if they fulfilled the 2000 American Thoracic Society (ATS)/European Respiratory Society (ERS) consensus criteria for IPF, which were slightly amended. First, basal and subpleural honeycombing on HRCT was required in patients not submitted to surgical lung biopsy (SLB), in keeping with the definition of definite pattern of usual interstitial pneumonia (UIP) of the 2018 updated consensus for the diagnosis of IPF. Second, SLB was mandatory for patients under 50 years of age. Only incident cases with a diagnosis established within 9 months before inclusion in the cohort were eligible. IPF diagnosis was adjudicated in a centralized multidisciplinary discussion. The enrolment period extended from 2007 to 2010, with a 5-year longitudinal prospective follow-up. The study was approved by the ethics committee and by the French data protection authority (CNIL: 908198).

Demographics, smoking status, clinical information, including history of comorbidities, and pulmonary function tests were obtained at inclusion. The COFI investigators were asked to provide information regarding patients' exposures to asbestos, crystalline silica, wood, organic, livestock, and metal dust. In addition, based on the investigator's judgment, patients could also have a job exposure assessment by an occupational health physician. However, hypersensitivity pneumonitis or pneumoconiosis were excluded by the COFI expert board and only IPF patients were included in COFI cohort.

Men and women were compared for characteristics at inclusion using *t*-test, Chi-squared or ANOVA, as appropriate. Survival was calculated between inclusion and death or transplantation. The survival probability of men and women was compared by a log-rank test. A Cox proportional hazards model was used for studying the survival after adjustment for potential confounders. Results of the Cox model are reported as hazard ratio (HR) and 95% confidence interval. Data were analyzed using R software V3.0.1.

## Results

Two hundred and thirty-six patients with new onset IPF were included in COFI cohort. The population consisted of 51 (22%) women and 185 (78%) men (Table 1). At inclusion, mean age was similar according to gender (70.1 ± 9.2 years in women vs. 67.4 ± 10.9 years in men, *p* = 0.08), but the proportion of women older than 65 was higher than in men (78 vs. 61%, *p* = 0.028). Seventy patients (30%) presented at least one occupational exposure (among asbestos, crystalline silica, wood, organic, livestock, and metal dust) without enough arguments for alternative diagnosis to IPF. Women significantly differed from men by a lower proportion of smokers (*p* < 0.001) and a lower frequency of occupational exposure (at least one exposure: 14 vs. 34% *p* = 0.012). At baseline, forced vital capacity (FVC) was greater in women than in men (83 ± 25 vs. 75 ± 19%, *p* = 0.046). Honeycombing (78.4 vs. 90.3%, *p* = 0.041) and emphysema (11.8 vs. 25.9%, *p* = 0.029) on computed tomography (CT) were less common in women. Comorbidities such as osteoporosis (9.8 vs. 2.2%, *p* = 0.025) and venous thromboembolic events (11.8 vs. 2.2%, *p* = 0.008) were more frequently reported among women.

**Table 1:** Characteristics of IPF patients included in COFI cohort according to gender.

**Patients with Idiopathic pulmonary fibrosis *N* = 246**	**Women *n* = 51**	**Men *n* = 185**	***P*-value**
**Age (years):** mean ± SD	70.1 ± 9.2	67.4 ± 10.9	0.08
**Age ≥ 65 years old:***n* (%)	40 (78%)	112 (61%)	**0.028**
**BMI:** mean ± SD	27.2 ± 4.70	27.5 ± 4.19	0.697
**Smoking history:***n* (%)			**<0.001**
Current	1 (2%)	15 (8%)	
Former	18 (35%)	131 (71%)	
Never	32 (63%)	39 (21%)	
**Occupational exposures:***n* (%)			**0.012**
At least one exposure^a^	7 (14%)	63 (34%)	
**Comorbidities:***n* (%)			
GastroEsophageal reflux	15 (29%)	52 (28%)	0.994
Ischemic heart disease	5 (10%)	36 (20%)	0.081
Arterial hypertension	31 (61%)	96 (52%)	0.161
Diabetes	9 (18%)	42 (23%)	0.559
Venous thromboembolic events	6 (12%)	4 (2%)	**0.008**
Osteoporosis	5 (10%)	4 (2%)	**0.025**
Sleep apnoea	3 (6%)	27 (15%)	0.157
**NYHA functional class at inclusion:***n* (%)			0.206
Class III–IV	16 (31%)	40 (22%)	
**Crackles at inclusion:***n* (%)	50 (98%)	175 (95%)	0.464
**Auto immune features at inclusion:***n* (%)			0.628
At least one positive auto antibody^b^	24 (47%)	77 (42%)	
**CT features at inclusion:***n* (%)			
Honeycombing	40 (78%)	167 (90%)	**0.041**
Emphysema	6 (12%)	48 (26%)	**0.029**
**PFTs at inclusion**			
TLC (ml): mean ± SD	3.238 ± 878	4.564 ± 1.110	**<0.001**
TLC (% predicted): mean ± SD	69.8 ± 18.3	69.1 ± 15.6	0.821
FVC (ml): mean ± SD	1.864 ± 572	2.820 ± 778	**<0.001**
FVC (% predicted): mean ± SD	83.0 ± 25.0	75.4 ± 18.7	**0.046**
FEV1(ml): mean ± SD	1.551 ± 487	2.327 ± 598	**<0.001**
FEV1 (% predicted): mean ± SD	84.3 ± 26.3	81.0 ± 19.4	0.406
DLCO (mmol/min/kPa): mean ± SD	3.81 ± 3.10	6.56 ± 7.20	**0.004**
DLCO (% predicted): mean ± SD	45.8 ± 15.1	48.3 ± 18.2	0.341
**6MWT at inclusion**			
Distance (m): mean ± SD	356 ± 132	428 ± 113	**0.001**
Distance (% predicted): mean ± SD	77.7 ± 27.7	80.5 ± 21.5	0.553
Desaturation <88%: *n* (%)	21 (53%)	84 (50%)	0.806
**Follow-up**			
Number of hospitalizations: mean ± SD	1.33 ± 1.90	1.12 ± 1.74	0.457
Acute exacerbation: *n* (%)	6 (12%)	27 (15%)	1.000
Death: *n* (%)	32 (63%)	104 (58%)	0.813
Transplantation: *n* (%)	1 (2%)	20 (11%)	**0.039**

a *asbestos, crystalline silica, wood, organic, livestock, and metal dust*.

b *Antinuclear antibodies, rheumatoid factor, myositis panel, and anti–cyclic citrullinated peptide, antineutrophil cytopasmic autoantibodies*.

*SD, Standard deviation; BMI, body mass index; CT, computed tomography; PFTs, pulmonary function tests; TLC, total lung capacity; FVC, forced vital capacity; FEV1, forced expiratory volume in 1 s; DLCO, diffusing capacity of the lung for carbon monoxide; 6MWT, six-minute walk test*.

*The values in bold are below the significance level of 0.05*.

**Figure 1:**
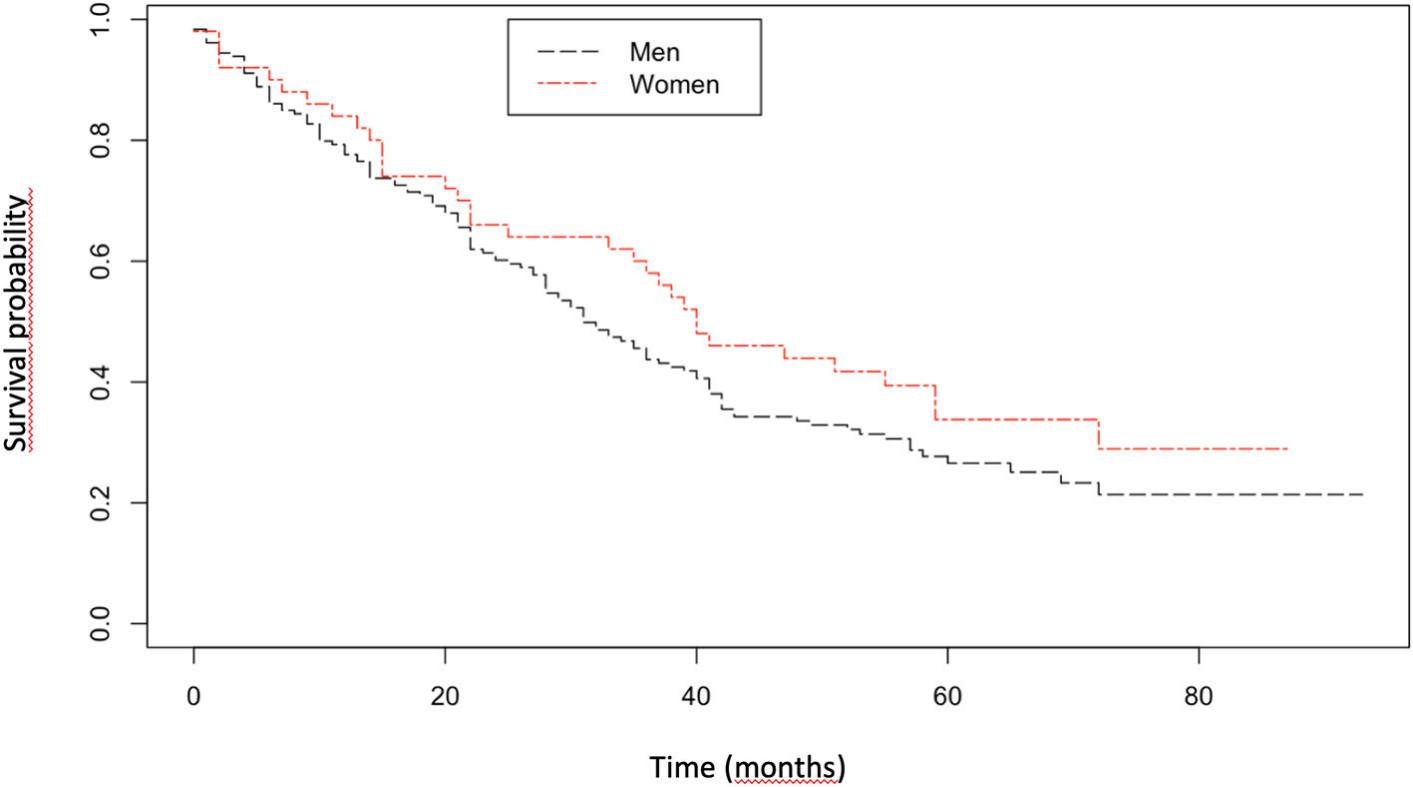
Kaplan–Meier survival curve for women and men with idiopathic pulmonary fibrosis.

The mean follow-up was 33.2 ± 23.6 months. At the end of study, 136 patients had died, 21 had a lung transplantation and 9 were lost to follow-up. Women were less likely to be transplanted (2 vs. 11%, *p* = 0.039). Median transplant-free survival was comparable between men and women (31 vs. 40 months, *p* = 0.2; Figure 1). After adjusting for age and FVC at inclusion, gender was not associated to a better transplant-free survival; and the survival HR was 0.85 [CI 95% (0.58–1.25), *p* = 0.41] for women. A sensitivity analysis was performed by taking into account the CT parameters (honeycombing and emphysema) for survival analysis and gender was not associated to a better transplant-free survival [HR was 0.77 (CI 95% (0.58–1.13), *p* = 0.18].

## Discussion

So far, only few studies have assessed IPF specificities according to gender. In the current study women had a different disease presentation at baseline, with a more preserved lung function and a different imaging pattern. Women were less likely to be transplanted but the median transplant-free survival was comparable in both genders.

In our cohort, women with IPF had less frequently exposure to inhaled aerocontaminants as compared to men. The differences in smoking history has been described previously, but this is the first study showing that other occupational exposures are more frequent in men than in women with IPF. These two types of exposures are strongly gender-related and their contribution in the development of IPF in women may increase in the future with changes in their life habits.

Furthermore, women with IPF seemed to have less severe disease at baseline, with a more preserved FVC. A better initial lung function in women has already been found in previous publications (3, 4). For the first time we demonstrate that honeycombing and emphysema on CT scan were more often observed in men as compared to women. This is consistent with a previous report suggesting that a surgical lung biopsy was required more frequently in women than in men for the diagnosis of IPF (5).

Interestingly, in our cohort, women were more likely than men to be over 65 years old. For various biological reasons, IPF may occur later in women than in men. On the other hand, as supported by our findings, men may develop IPF earlier because they are more exposed to fibrotic triggers such as tobacco smoke or occupational agents. Conversely, in another study, women were younger at IPF diagnosis, so further clarification is needed (6).

In keeping with previous publications, IPF men were more likely to undergo lung transplantation, probably because in our cohort, women were older, with more comorbidities (4, 6). Contrary to several studies indicating a better survival in women (4, 6–8) we didn't find any significant differences in survival between women and men. We probably missed differences in survival between gender due to the small number of patients and a lack of power. The stringent inclusion criteria used in COFI cohort maybe selected a more homogenous population of IPF than patients from other studies. However, better survival in IPF women may be emphasized in studies using IPF code-based diagnostic (like ICD9 516.3 ok ICD 10 J84.1) which can capture other idiopathic ILDs like non-specific pneumonitis (NSIP).

On the other hand, two national registries showed, that over the period 1999–2017, the mortality rate increased more among IPF women compared to men (9, 10). Nevertheless, the percentage of women in our cohort was slightly lower than that generally reported in the literature, and our results on survival may be due to a small number of patients and a lack of power.

In conclusion, as compared to men, women appear to be older, to have less frequently a history of smoking or occupational exposures, to have a different imaging pattern and more preserved lung function at diagnosis of IPF, and to have less recourse to lung transplantation, however with comparable survival.

## Data Availability Statement

The raw data supporting the conclusions of this article will be made available by the authors, without undue reservation.

## Ethics Statement

The studies involving human participants were reviewed and approved by ethics committee and by the French data protection authority (CNIL: 908198). The patients/participants provided their written informed consent to participate in this study.

## Author Contributions

YU and DV: conceptualization. LS: formal analysis. DV: funding acquisition. DV, ZC, HN, VC, DI-B, BC, SG-D, JC, BW, AT, BM, GP, YU, SM-A, SH, SD, VG, AG, PB, JT, KJ, RB, OF, TG, and CP: investigation. YU, LS, HN, DV, CP, TG, and OF: methodology. DV, ZC, HN, VC, DI-B, BC, SG-D, JC, BW, AT, BM, GP, YU, SM-A, SH, SD, VG, AG, PB, JT, KJ, RB, OF, TG, and CP: resources. YU, HN, and DV: supervision. YU: validation. LS, YU, VC, HN, and DV: writing-original draft. LS and YU: writing-review and editing. All authors contributed to the article and approved the submitted version.

## Conflict of Interest

LS reports personal fees and non-financial support from Roche/Genentech, non-financial support from Boehringer Ingelheim, personal fees from Boehringer Ingelheim, outside the submitted work. HN reports grants and personal fees from Roche/Genentech, Boehringer Ingelheim, personal fees from Galapagos, other from Sanofi, Gilead, Novartis, Galecto Biotech AB, during the conduct of the study; personal fees from Actelion Pharmaceuticals, outside the submitted work. VC reports personal fees and non-financial support from Actelion, grants, personal fees and non-financial support from Boehringer Ingelheim, personal fees from Bayer/MSD, Novartis, personal fees and non-financial support from Roche/Promedior, personal fees from Sanofi, Celgene, Galapagos, Galecto, Shionogi, Astra Zeneca, Fibrogen, outside the submitted work. BC reports personal fees from Astra Zeneca, grants, personal fees and non-financial support from Boehringer Ingelheim, BMS, personal fees from Sanofi, grants, personal fees and non-financial support from Roche, outside the submitted work. AT reports personal fees from Chiesi, other from VitAlaire, Astrazeneca, Boehringer Ingelheim, outside the submitted work. SM-A reports other from roche, other from Boehringer Ingelheim, outside the submitted work. SH reports personal fees from Roche, Boerhinger ingelheim, grants from CSL Behring, outside the submitted work. SD reports personal fees from Boehringer-Ingelheim, Chiesi, Roche, outside the submitted work. PB reports personal fees and other from Roche, Boehringer, Novartis, personal fees from TEVA, other from Chiesi, personal fees from AstraZeneca, other from Stallergene, outside the submitted work. RB reports grants and personal fees from Roche, Boerhinger Ingelheim, personal fees from Savara, outside the submitted work. CP reports personal fees from ROCHE, outside the submitted work. DV reports personal fees from Roche, Boehringer Ingelheim, Roche & BI, outside the submitted work. YU reports personal fees and non-financial support from Boehringer, Roche, grants, personal fees and non-financial support from Oxyvie, outside the submitted work. The remaining authors declare that the research was conducted in the absence of any commercial or financial relationships that could be construed as a potential conflict of interest.

## Publisher's Note

All claims expressed in this article are solely those of the authors and do not necessarily represent those of their affiliated organizations, or those of the publisher, the editors and the reviewers. Any product that may be evaluated in this article, or claim that may be made by its manufacturer, is not guaranteed or endorsed by the publisher.
